# Cutaneous Histoplasmosis in Advanced HIV: A Case of Systemic Fungal Dissemination

**DOI:** 10.7759/cureus.110332

**Published:** 2026-06-05

**Authors:** Diana S Blanquet Campos, Frida M Ortega García, Lorena Morfin Hernandez, Mónica S Garcia Bravo, Verónica González Sánchez, Martha A Aceves Villalvazo

**Affiliations:** 1 Department of Internal Medicine, Hospital Regional “Dr. Valentín Gómez Farías” Instituto de Seguridad y Servicios Sociales de los Trabajadores del Estado (ISSSTE), Zapopan, MEX; 2 Department of Dermatology, Hospital Regional “Dr. Valentín Gómez Farías” Instituto de Seguridad y Servicios Sociales de los Trabajadores del Estado (ISSSTE), Zapopan, MEX

**Keywords:** disseminated histoplasmosis, histoplasma capsulatum, hiv, syphilis, tuberculosis

## Abstract

Disseminated histoplasmosis is a severe opportunistic infection in patients with human immunodeficiency virus (HIV) infection, particularly in those with CD4 lymphocyte counts of ≤50 cells/µL. Cutaneous involvement usually occurs as part of disseminated disease and, in some cases, may represent the first manifestation of systemic involvement. Cutaneous manifestations are highly variable, and these lesions are commonly associated with constitutional symptoms and pulmonary involvement.

We present the case of a 53-year-old man with a history of HIV infection clinical stage C3, miliary tuberculosis with incomplete treatment, and poor therapeutic adherence. He developed a pruritic dermatosis characterized by erythematous lesions associated with fever, odynophagia, asthenia, and adynamia. At admission, generalized and symmetrical dermatosis was observed, consisting of innumerable erythematous-violaceous papules and macules coalescing into plaques, in addition to palmoplantar lesions with a collarette scale resembling the “Biett sign.” Histopathological examination demonstrated histiocytes filled with multiple intra- and extracellular yeast-like structures consistent with *Histoplasma capsulatum*. HIV viral load was reported at 8,070 copies/mL and CD4 T-lymphocyte count at 46 cells/µL. Based on these findings, a diagnosis of disseminated cutaneous histoplasmosis was established. During hospitalization, the patient received liposomal amphotericin B, and upon discharge, antiretroviral therapy (ART) was initiated, resulting in favorable clinical evolution and the improvement of the cutaneous lesions during outpatient follow-up.

This case illustrates the diagnostic complexity of disseminated cutaneous histoplasmosis in patients with advanced immunosuppression and highlights the value of skin biopsy and early histopathological identification in establishing a timely diagnosis and initiating prompt antifungal treatment.

## Introduction

Disseminated histoplasmosis is a severe opportunistic infection that affects approximately 5%-10% of cases, particularly in human immunodeficiency virus (HIV) infection with advanced immunosuppression and CD4 lymphocyte counts of ≤200 cells/µL. Its clinical presentation is often nonspecific, which complicates timely diagnosis and delays the initiation of antifungal treatment, contributing to high mortality rates. In this context, cutaneous involvement is frequent and generally occurs as part of disseminated disease, as previously mentioned in those with <200 cells/µL but particularly in CD4 counts of <50 cells/µL who are not receiving antiretroviral therapy (ART), and may represent the first manifestation of systemic involvement [1-4].

Cutaneous manifestations are highly variable and include papules, nodules, infiltrated plaques, ulcers, and lesions mimicking other infectious or inflammatory dermatoses, making differential diagnosis challenging [2-5]. These lesions are commonly associated with constitutional symptoms such as fever, weight loss, and pulmonary involvement. Although dermoscopy may reveal suggestive findings, these lack specificity and may overlap with other deep mycoses [1,4].

The diagnosis should be suspected in patients with advanced HIV infection presenting with atypical skin lesions accompanied by systemic symptoms. The detection of *Histoplasma* antigen in blood or urine constitutes the most sensitive diagnostic test, whereas the culture and biopsy of affected tissues allow diagnostic confirmation [6,7].

The treatment of choice consists of liposomal amphotericin B, followed by itraconazole. The early initiation of antifungal therapy, together with timely antiretroviral treatment, is essential to improve prognosis and reduce mortality [5-9].

## Case presentation

A 53-year-old man from Puerto Vallarta, México, with a history of chronic substance abuse, including cocaine and methamphetamine use since youth, with last reported use 15 days prior to hospitalization, as well as alcohol and tobacco use, presented to our institution. His infectious history was significant for HIV infection diagnosed in May 2024, classified as clinical stage C3, for which antiretroviral therapy with emtricitabine and tenofovir had been prescribed but poorly adhered to. Subsequently, he was diagnosed with miliary tuberculosis and prescribed antifimic therapy, which he also discontinued without attending follow-up appointments.

**Figure 1:**
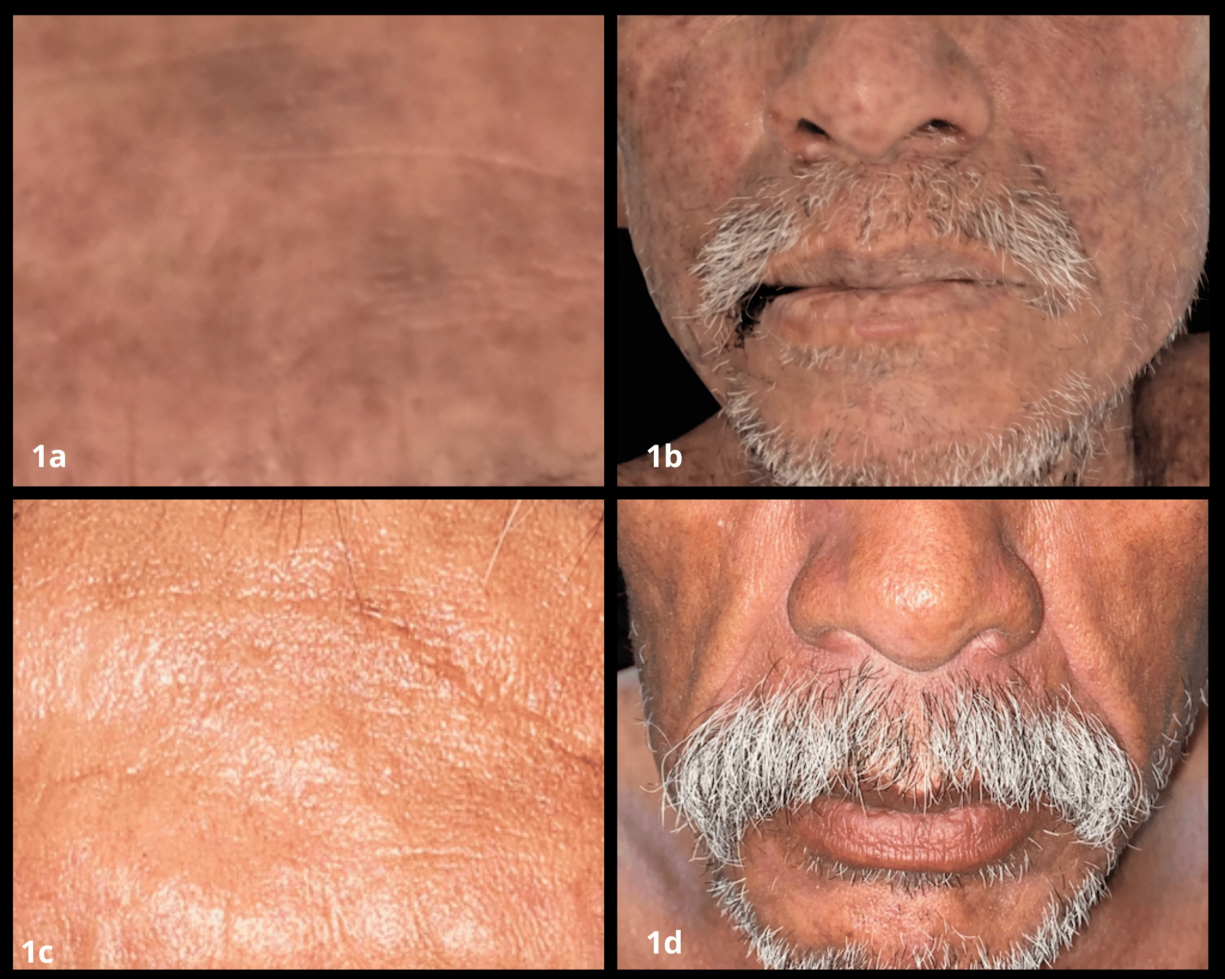
Dermatological lesions on the face (1a and 1b) Generalized dermatosis involving the facial region consisting of 2-3 mm erythematous-violaceous plaques with well-defined borders, symmetrically distributed, with apparent chronic evolution. (1c and 1d) Comparative photographs obtained six months after the initial presentation, where the improvement of the lesions is evident

**Figure 2:**
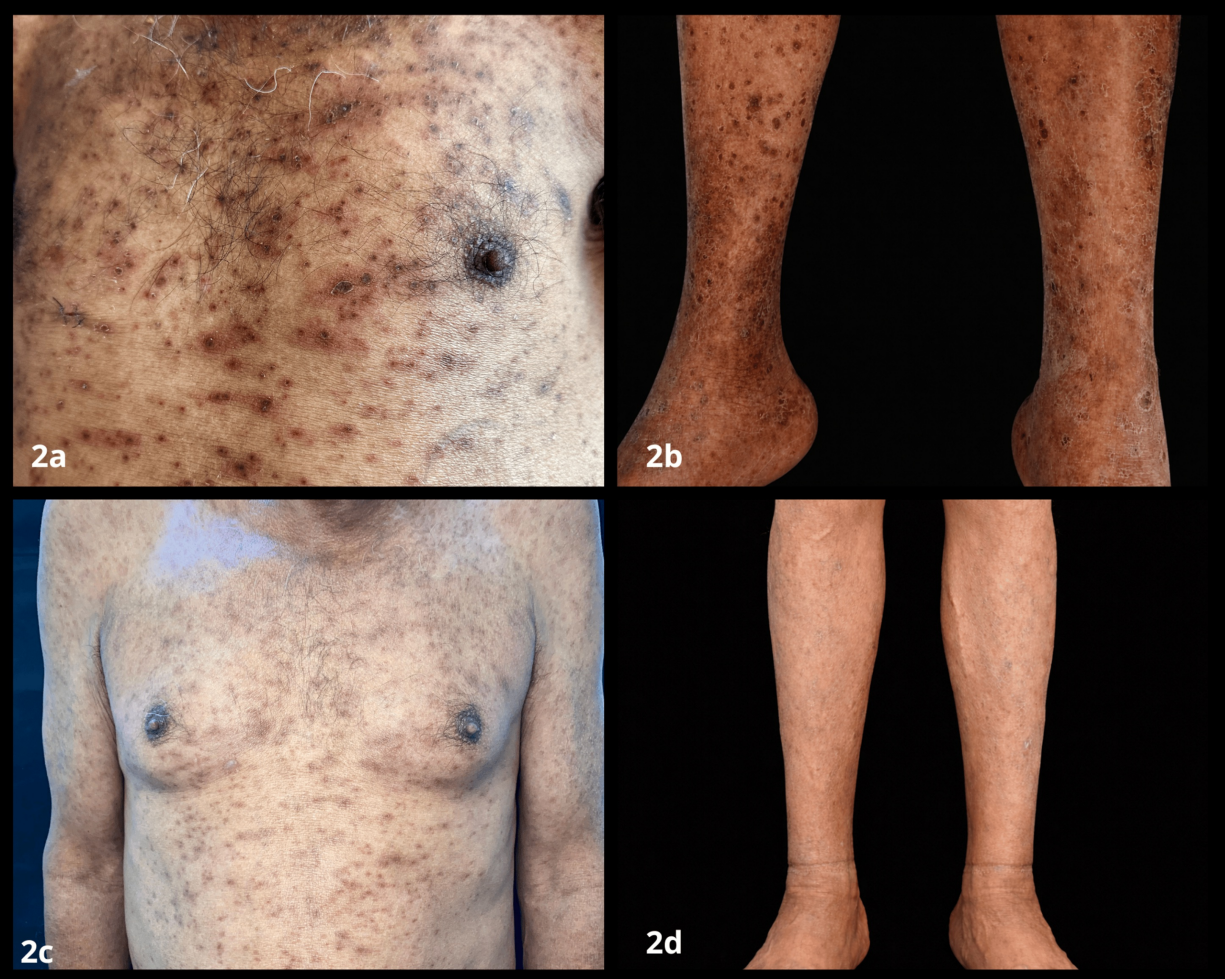
Dermatological lesions in lower extremities (2a and 2b) Generalized dermatosis involving lower extremities, consisting of 2-3 mm erythematous-violaceous plaques with well-defined borders, symmetrically distributed, with apparent chronic evolution. (2c and 2d) Comparative photographs obtained six months after the initial presentation, demonstrating improvement in the previously described regions, with the decreased pigmentation of the erythematous-violaceous plaques

**Figure 3:**
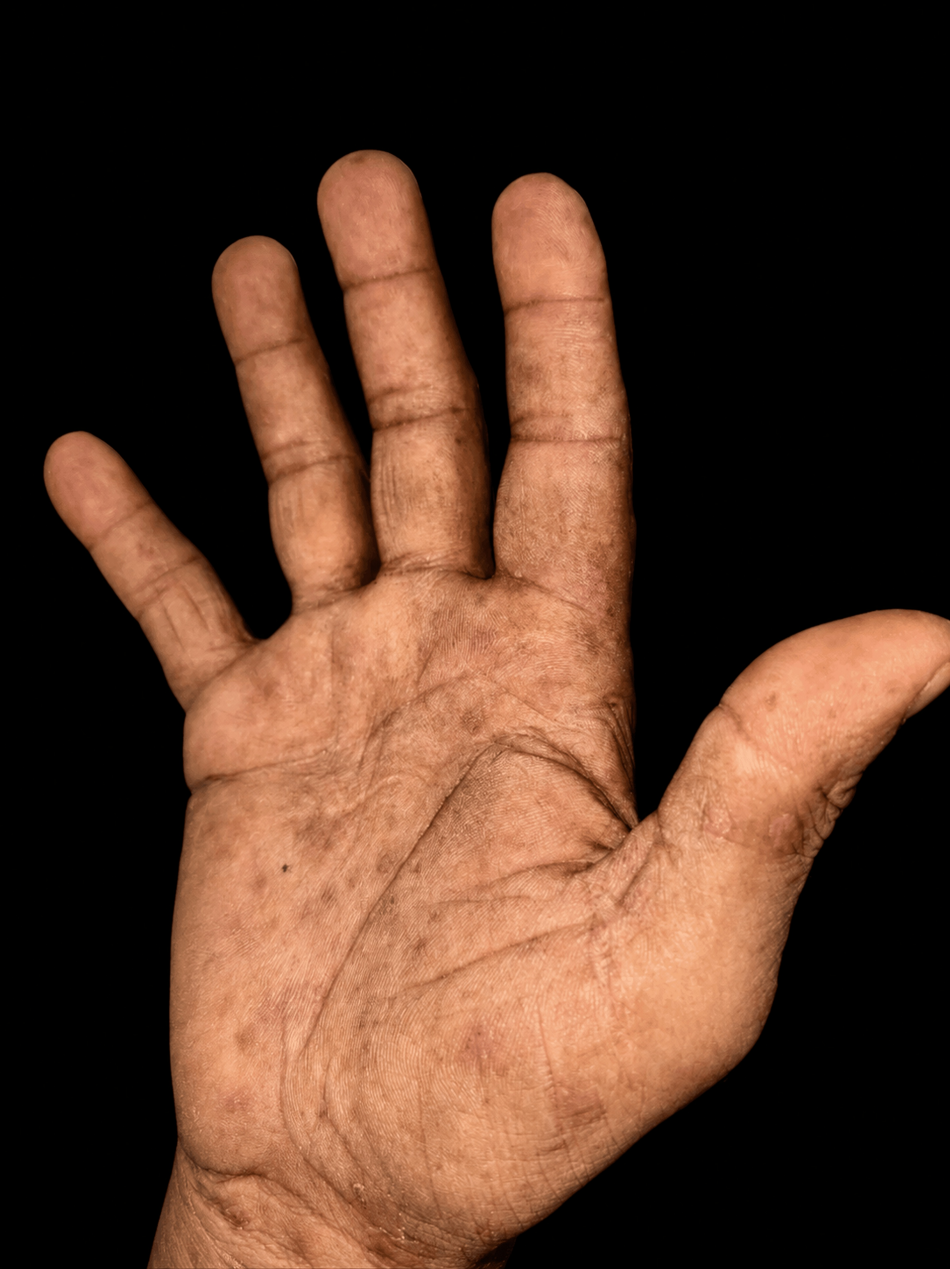
Dermatological lesions on the patient’s hand Hyperpigmented macules with a thin white collarette scale resembling the “Biett sign” were also observed on the palms and soles

The current illness began approximately two months prior to admission with a pruritic dermatosis characterized by erythematous lesions on the upper extremities that progressively spread to the trunk, face, and mucous membranes, eventually becoming generalized and involving the palms and soles. The patient reported a previous single genital lesion that was nonspecific, asymptomatic, and self-limited. The clinical picture was accompanied by fever up to 40°C, odynophagia, asthenia, adynamia, and burning pain in the extremities rated 10/10 on the visual analog scale (VAS), as well as episodes of generalized involuntary movements without the loss of consciousness or sphincter control. Upon admission to the internal medicine department, septic shock was documented, and vasopressor support was initiated through a central venous catheter. A nasojejunal tube was placed for nutritional support and a Foley catheter for strict fluid monitoring. Neurological examination revealed a tendency toward hyporeactivity, without focal deficits or supplemental oxygen requirement. Dermatological examination showed dermatosis involving the head and neck (Figure 1a, 1b), trunk, and upper and lower extremities, including palms and soles, as well as oral and genital mucosa. The lesions were generalized and symmetrical, consisting of innumerable millimetric erythematous-violaceous papules and macules coalescing into larger plaques with well-defined borders, some with surface scaling and chronic excoriations (Figure 2a, 2b). Hyperpigmented macules with a thin white collarette scale resembling the “Biett sign” were also observed on the palms and soles (Figure 3).

**Figure 4:**
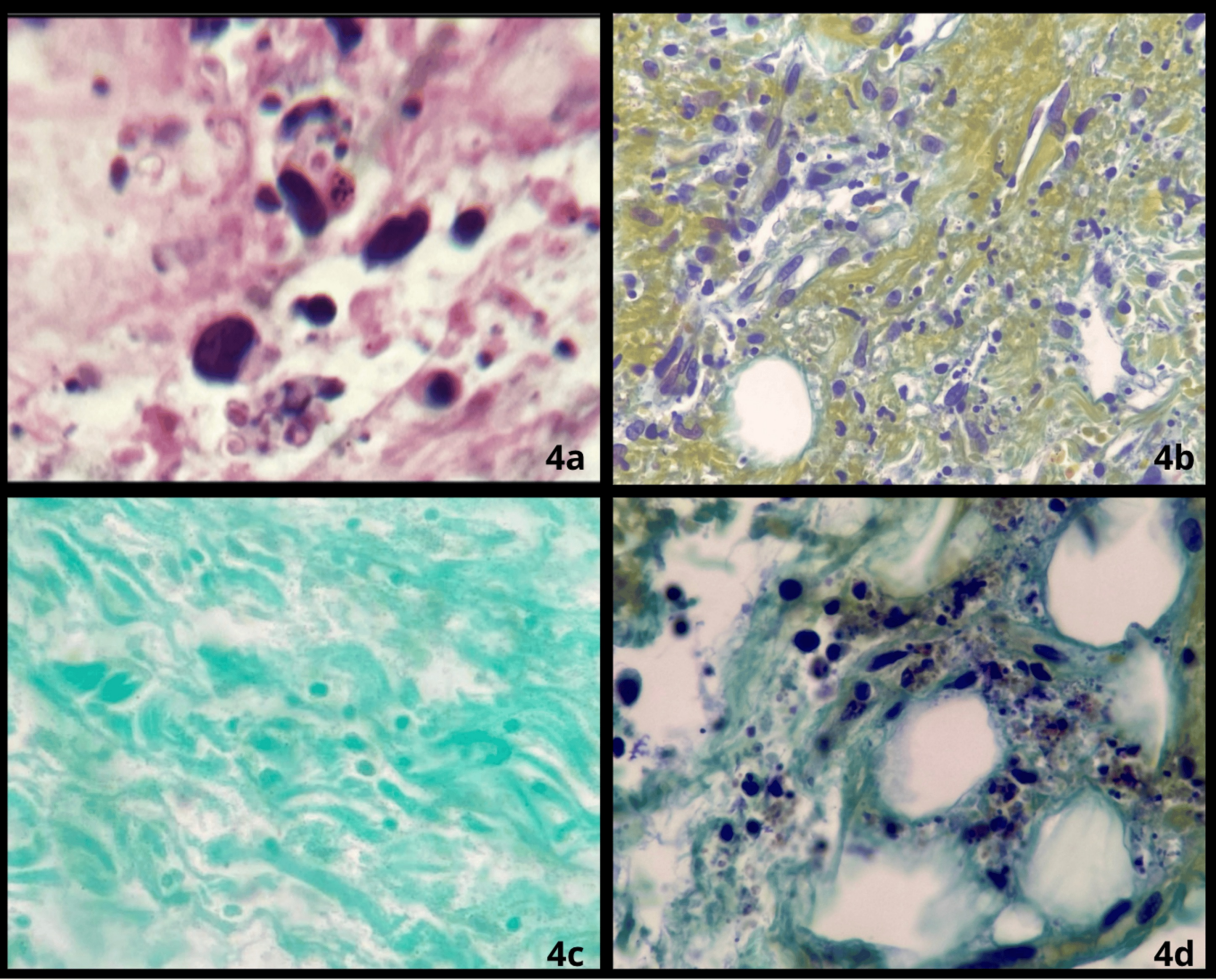
Biopsy (4a) H&E staining (100×) showing a dermal infiltrate predominantly composed of epithelioid histiocytes and lymphocytes. Histiocytes containing multiple small round-to-oval yeast-like structures measuring approximately 2-5 µm in diameter are observed, distributed both intracellularly within macrophages and extracellularly, although to a lesser extent. The yeasts exhibit thin walls, scant cytoplasm, and narrow-based budding. In some fields, a clear perinuclear halo is observed. (4b and 4c) Grocott (40×) and modified Grocott staining (20×). (4d) Giemsa and toluidine blue staining (100×), confirming the previously described structures

Three biopsies were obtained by puncture: one for bacteriological culture with India ink staining that was subsequently reported as having no growth, another for culture on Sabouraud agar, and the last one for histopathological analysis. Pathology report (Figure 4a) described a dermal infiltrate composed predominantly of epithelioid histiocytes and lymphocytes. Histiocytes contained multiple small round-to-oval yeast-like structures measuring approximately 2-5 µm in diameter, distributed intracellularly within macrophages and, to a lesser extent, extracellularly. The yeasts exhibited thin walls, scant cytoplasm, and narrow-based budding. In some fields, a clear perinuclear halo was observed. These findings correlated with the presence of intra- and extracellular fungal structures consistent with *Histoplasma capsulatum*. Grocott and modified Grocott staining (Figure 4b, 4c), as well as Giemsa with toluidine blue staining (Figure 4d), demonstrated the same previously described structures. Lumbar puncture was also performed; cerebrospinal fluid analysis showed nonspecific cytochemical findings, negative bacteriological culture, and negative PCR for *Mycobacterium tuberculosis*. Although Venereal Disease Research Laboratory (VDRL) testing in cerebrospinal fluid could not be performed, serum Venereal Disease Research Laboratory (VDRL) testing was negative on two occasions; however, highly specific treponemal tests were unavailable to confirm these findings. Chest computed tomography revealed bilateral ground-glass opacities and multiple disseminated nodules compatible with a pneumonic process, although sputum samples and bronchoalveolar lavage could not be obtained. Immunovirological studies reported an HIV viral load of 8,070 copies/mL and a CD4 T-lymphocyte count of 46 cells/µL. QuantiFERON-TB Gold testing was indeterminate, likely related to the patient’s severe immunosuppression.

*Histoplasma* antigen testing and fungal cultures from the skin lesions could not be performed because these diagnostic resources were unavailable at our institution. Based on the clinical, immunological, and histopathological findings, a diagnosis of disseminated cutaneous histoplasmosis was established in a patient with stage C3 HIV infection, severe immunosuppression, and a previous history of miliary tuberculosis who presented in septic shock. During hospitalization, vasopressor support was successfully discontinued, and the patient received liposomal amphotericin B at a dose of 3 mg/kg/day for three weeks, giving a total of 150 mg per day calculated at a weight of 50 kg. Given the profound CD4+ T-lymphocyte depletion, prophylactic treatment with trimethoprim-sulfamethoxazole and ganciclovir was initiated; however, the latter was subsequently discontinued due to a lack of a precise indication.

The patient demonstrated favorable clinical improvement, allowing hospital discharge with outpatient follow-up by the infectious diseases, internal medicine, and dermatology services. Six months later, comparative photographs obtained during follow-up evaluation of the face (Figure 1c, 1d) and the remaining body regions (Figure 2c, 2d) showed marked clinical improvement, characterized by reduced pigmentation of the previously described lesions and the absence of new opportunistic infections. Antiretroviral therapy and prophylactic treatment were reinitiated according to the patient’s immunological status and CD4+ T-lymphocyte count. Regarding the previously reported incomplete antifungal therapy, treatment adherence was reassessed and confirmed to be at least 80%, corresponding to approximately five months of the prescribed regimen. Therefore, additional antifungal treatment was not considered necessary. One month after hospital discharge, the patient attended an infectious diseases follow-up visit, during which antiretroviral therapy was restarted with bictegravir, emtricitabine, and tenofovir alafenamide. Prophylaxis with trimethoprim-sulfamethoxazole was continued.

## Discussion

This case illustrates the complexity of managing patients with advanced HIV infection characterized by profound immunosuppression, poor therapeutic adherence, and the coexistence of opportunistic infections. The generalized and florid cutaneous presentation accompanied by systemic involvement initially suggested differential diagnoses such as secondary syphilis, drug reactions, cutaneous tuberculosis, and dermatoses associated with immunosuppression; however, the histopathological identification of *Histoplasma capsulatum* confirmed the diagnosis of disseminated cutaneous histoplasmosis [1,4,5].

Although cerebrospinal fluid VDRL testing could not be performed, non-treponemal serologic testing for syphilis (serum VDRL) was negative on two occasions, the maximum dilution that could be performed was 1:64 to rule out a prozone effect; unfortunately, this is the only means available in our hospital setting since we do not have complementary treponemal tests. Nevertheless, specific treponemal tests were unavailable to definitively confirm or exclude infection. In this context, the possibility of a false-negative result could not be ruled out, particularly due to the prozone phenomenon described in patients with high antibody titers and advanced immunosuppression. Therefore, coinfection with secondary syphilis cannot be completely excluded, especially considering that the initial lesion described by the patient could be compatible with a primary chancre. Additionally, literature reports that cutaneous histoplasmosis may present with lesions clinically mimicking secondary syphilis, increasing diagnostic difficulty [1,4].

The unfavorable clinical course was conditioned by severe immunosuppression, evidenced by a CD4 T-lymphocyte count of <50 cells/µL, as well as poor adherence to both antiretroviral and antifungal therapies, factors that favored disease progression and the possible coexistence of multiple infectious processes [1,6,9]. This case underscores the importance of considering histoplasmosis in the differential diagnosis of patients with HIV presenting with disseminated cutaneous lesions, particularly in endemic regions or in patients with exposure history, as well as the need for a comprehensive approach including timely diagnosis, adequate antifungal treatment, the initiation or reintroduction of antiretroviral therapy (ART), and close follow-up [5,7].

Histoplasmosis may present as pulmonary, chronic, or disseminated disease. In this case, although the microbiological confirmation of the etiologic agent responsible for the pneumonic process could not be obtained, severe immunosuppression and subsequent cutaneous dissemination suggest that *Histoplasma capsulatum* may have been responsible for the initial pulmonary involvement [1,2]. Regarding cutaneous manifestations, the clinical presentation was most consistent with secondary cutaneous histoplasmosis, an entity associated with advanced immunosuppression, particularly in patients with HIV, occurring in up to 10%-25% of cases, which usually originates from a primary pulmonary focus and is accompanied by ulcerative lesions, as occurred in this patient [8,9].

## Conclusions

In conclusion, this case highlights the critical diagnostic value of skin biopsy in patients with advanced immunosuppression, where disseminated histoplasmosis may present with polymorphic cutaneous manifestations that mimic other infectious or inflammatory dermatoses. In such settings, histopathological evaluation can provide a rapid and reliable diagnosis, allowing for the timely initiation of appropriate antifungal therapy. Early recognition, combined with multidisciplinary management and adherence to both antiretroviral and antifungal treatment, remains essential for improving clinical outcomes and reducing the substantial morbidity and mortality associated with HIV-related disseminated histoplasmosis.
